# Community intervention study of viscera massage in overweight/obese type 2 diabetes high-risk population

**DOI:** 10.1097/MD.0000000000027932

**Published:** 2021-12-03

**Authors:** Gaofeng Wang, Deyu Cong, Hongyu Ju, Jiabao Sun, Chaozheng Li, Zepeng Zhang, Yunjie Chu, Xingquan Wu

**Affiliations:** a College of Acupuncture and Tuina, Changchun University of Chinese Medicine, Changchun, Jilin, China; b Department of Health Management, Baicheng Medical College, Baicheng, Jilin, China; c Department of Tuina, Affiliated Hospital of Changchun University of Chinese Medicine, Changchun, Jilin, China; d Department of Endocrine and Metabolic Diseases, Baicheng Municipal Hospital, Baicheng, Jilin, China; e College of Traditional Chinese Medicine, Changchun University of Chinese Medicine, Changchun, Jilin, China.

**Keywords:** abdominal massage, community intervention study, gut microbiota, prediabetes, viscera massage

## Abstract

**Background:**

Prediabetes is an intermediate metabolic state between normoglycemia and diabetes. Without intervention, prediabetes often progresses to diabetes and prediabetes is associated with increased risk of cardiovascular disease, cancer, renal disease, and dementia. Lifestyle modification play a major role in controlling prediabetes. But lifestyle interventions are often with poor compliance and side effects of drugs are often be dislike by people. As a non-invasive therapy with no side effects, abdominal massage (AM), also called viscera massage in China, has been used to treat prediabetes and obesity-associated diseases. The gut microbiota has been recognized as an important factor in the development of metabolic diseases. Individuals with prediabetes have aberrant intestinal microbiota character. Colonic transport time and stool consistency are strongly associated with gut microbiota. Viscera massage can ease constipation by reducing colonic transport time and promoting intestinal motility. We can infer that viscera massage can modulate composition of gut microbiota affects human metabolism. So, in this trial, we will explore the mechanism of viscera massage on prediabetes from the perspective of intestinal microbiota.

**Methods and design:**

Eighty prediabetes individuals will be recruited for this study. Eighty prediabetes individuals will be divided into lifestyle intervention group and viscera massage + lifestyle intervention group by a simple random method. Each group will have 40 individuals. The manipulation of the viscera massage + lifestyle intervention group will be mainly carried out through rubbing the abdomen, kneading abdomen, vibrating abdomen, and pressing the abdomen, 30 minutes per time, once a day, with 2 days off every 5 days. Lifestyle interventions will be performed by combining pushing healthy lifestyle guidance information through Wechat application and giving face-to-face advice together daily. The lifestyle intervention group will receive healthy lifestyle intervention only. All the intervention will be conducted for 4 weeks. Weight, body mass index (BMI), waist circumference, waist-to-hip ratio, and waist-to-height ratio will be measured at the last day of every week. Triglycerides, total cholesterol, low-density lipoprotein cholesterol, high-density lipoprotein cholesterol, fasting blood-glucose, 2-hour post-meal blood glucose (2hPG) and glycosylated hemoglobin, fasting insulin and insulin resistance index will be tested at the first day and last day of the intervention course. The fecal samples of subjects will be gathered at the first day and last day of the intervention course and will be performed 16S rRNA gene sequencing and metagenomic detection. Finally, the effect and potential mechanism of viscera massage on prediabetes will be discussed in combination with all the results.

**Discussion:**

The results of this study will be used to verify the effect of AM on prediabetes and explore the mechanism of AM on prediabetes from the perspective of gut microbiota.

## 1 Introduction

Prediabetes is an intermediate metabolic state between normoglycemia and diabetes, including impaired fasting glucose (IFG), impaired glucose tolerance (IGT), and the mixed-status (IFG+IGT).^[1]^ Approximately 5–10% of people with prediabetes will become diabetic each year, and up to 70% of individuals with prediabetes will eventually develop diabetes in their lifetime.^[2]^ The U.S. National Health and Nutrition Examination Survey suggests that 35% of US adults over 20 years of age and 50% of those over 65 had prediabetes in 2005 to 2008 based on fasting glucose or glycosylated hemoglobin (HbA1c) levels.^[3]^ Within the Asian region, the prevalence of prediabetes among adults reaches 50% in China, with Indians 18.9%, and Malays 22.6% respectively.^[4,5]^ Prediabetes is more prevalent than diabetes^[6]^ and pre-diabetes is more prevalent in women (21.9%) and urban dwellers (21.5%).^[4]^ Without intervention, prediabetes often progresses to diabetes and prediabetes is associated with increased risk of cardiovascular disease, cancer, renal disease, and dementia.^[7]^ People with prediabetes may have concomitant damage to end organs, such as eyes, kidneys, blood vessels, and the heart that are traditionally considered to be complications of diabetes.^[3]^ Lifestyle modification, including weight loss, dietary changes, and increased physical activity, play a major role in controlling prediabetes. Significant evidence also supports the effectiveness of a combination of lifestyle modification and pharmacologic therapy, such as metformin, in delaying the onset of diabetes.^[4]^ But lifestyle interventions are often challenging and with poor compliance.^[8]^ People are always afraid of the side effects of drugs. As a non-invasive treatment with no side effects, AM, also called viscera massage in China, has been used to treat prediabetes as well as the associated overweight and obesity. Mechanical AM and manual AM had been used to reduce cellulite,^[9]^ thigh and infraumbilical circumference,^[9]^ arm and post-partum abdominal, and flank subcutaneous fat deposits,^[10]^ improve skin laxity,^[10]^ and serum lipids.^[11]^ Manual AM also can enhance the effectivity of cryolipolysis treatment.^[12]^ As prediabetic individuals are often overweight or obese,^[13]^ AM has been used to manage prediabetes, and can significantly reduce the levels of fasting blood-glucose (FBG) and 2 hours postprandial blood glucose (2hPBG) in patients with prediabetes, with the decrease of 2hPBG more significant.^[14]^ In order to uncover the underlying mechanism of AM, several animal experiments had been carried out. AM can alleviate glucose and lipid metabolism disorder by regulating of AMPK/Glut4 signaling chain, the gene transcription and protein expression of Nuclear factor kappa beta and interleukin-6 in skeletal muscle,^[15,16]^ gut microbiota and bile acid metabolism^[17]^ in T2DM rats. Prediabetes is intertwined with the increasing incidence of metabolic syndrome and obesity. Prediabetes and T2DM has similar pathophysiology. However, there are few clinical studies and mechanism studies specially on prediabetes.

The gut microbiota has been recognized as an important factor in the development of metabolic diseases such as prediabetes, T2DM, and obesity, and is considered an endocrine organ involved in the maintenance of energy homeostasis and host immunity.^[18]^ The intestinal microbiota species may interact with host metabolism through metabolite-mediated stimulation of enteric hormones and other systems outside of the gastrointestinal tract, such as the endocannabinoid system. Individuals with prediabetes have aberrant intestinal microbiota characterized by a decreased abundance of the genus Clostridium and the decreased abundance of bacterium *A. muciniphila*.^[19]^ Based on previous studies and the important role of gut microbiota in metabolism, the proposed clinical trial will determine the possible effects of AM on prediabetes by evaluating the FBG, 2-hour post-meal blood glucose (2hPG), HbA1c, fasting insulin, insulin resistance index (HOMA-IR), blood lipid, BMI, waist-to-hip ratio, and waist-to-height ratio (WHtR), and will explore the mechanism of viscera massage on prediabetes from the perspective of intestinal microbiota.

## 2 Methods and design

### 2.1 Objectives

To determine the possible effects of viscera massage on prediabetes and to investigate the role of gut microbiota in improving prediabetes.

### 2.2 Setting

The patients will be recruited from the Department of tuina, Affiliated Hospital of Changchun University of Chinese Medicine, Hongqi First Community Health Service Center, Chaoyang District and Hongqi Second Community Health Service Center, Chaoyang District. Patients participating in the trial will be screened strictly according to the diagnostic criteria, inclusion criteria, and exclusion criteria. The sample size estimation will be based on the clinical calculation formula employed for small sample size. The estimate sample size is (n = 36). So, the therapeutic effect could be evaluated by the sample size of >36 in each group. In this study, 80 individuals will be recruited by simple random method and divided into two groups, 40 individuals in each group.

The researcher of the project will record the subject's basic information (such as date of birth, age, sex, and address) and answer questions patient involved in the trial. After completed the recruitment, individuals involved will be assigned a trial study serial number, also the patient's unique identification number, according to the order of enrollment, until the total numbers (n = 80) are all allocated. The odd-numbered individual was divided into the lifestyle intervention group, with the even-numbered individual the viscera massage + lifestyle intervention group. After clinical evaluation, blood and stool sampling, viscera massage therapy will be adopted.

### 2.3 Participants and recruitment

**Figure 1:**
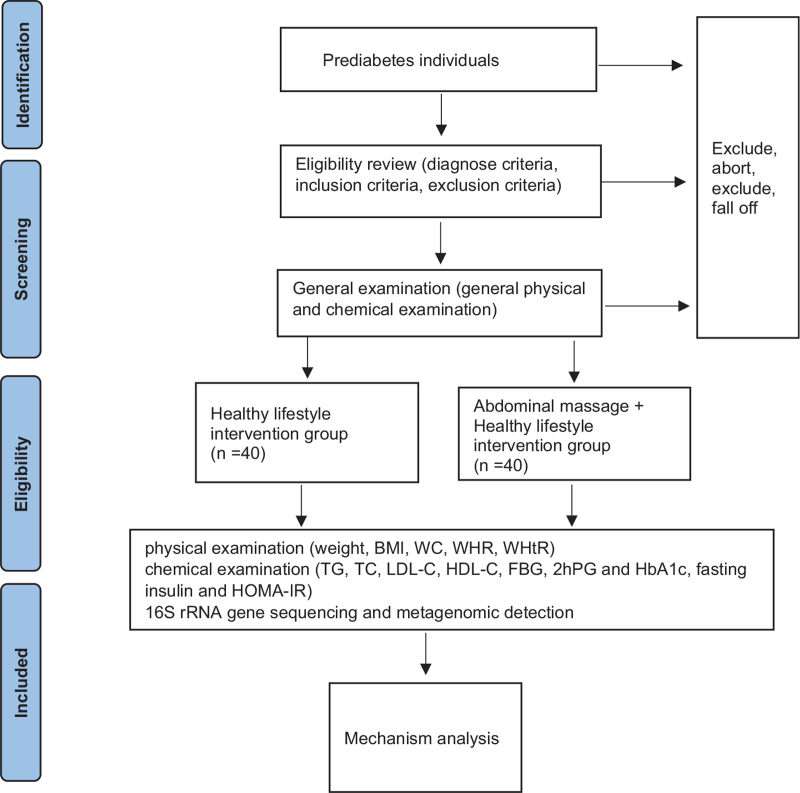
Trial flow chart.

The recruitment posters will be displayed in the hospitals and community. And the forward recruitment information will be posted on WeChat to recruit the potential participants for this study. The participants will be explained in detail about the potential benefits and services to be provided and the risks associated with participating in the study, including the possible associated adverse clinical outcomes with viscera massage therapy. An informed consent will be signed after reading carefully by each participant prior to the participation. On the consent form, participants will also be asked whether they agree to use their data even if they opt out of the trial. The participants will also be required to obtain permission for the research team to share relevant information with staff from the participating hospitals or regulatory agencies. The participants will receive 4 different courses of viscera massage therapy. The personal information of all the participants will be kept confidential by the researchers and used only for this study. The patients must properly understand and sign informed consent. The FBG and 2hPG will be used to potentially determine whether participants meet the inclusion criteria. Patients who met the criteria will be included in the study (Fig. 1).

### 2.4 Ethical issues

This research project has been reviewed and approved by The Ethics Committee of the Affiliated Hospital of Changchun University of Chinese Medicine (CCZYFYLL2021 Standard word -002). This project was funded by National Key Research and Development Program (2019YFC1709904), Education Department of Jilin Province (JJKH20210959KJ), Health and Health Technology Innovation Project of Jilin Province (2020J065), Postdoctoral Research program of Jilin Province ([2020]498), and Science and Technology Development Plan of Jilin Province (20200403124SF).

### 2.5 Diagnostic criteria

The diagnostic criteria refer to “Intervention for adults with prediabetes: A Chinese expert consensus.”^[20]^ The participants will have to meet the following criteria: the blood level of IFG should be at 6.1 mmol/L < FPG < 7.0 mmol/L and 2hPG < 7.8 mmol/L, the blood level of IGT should be at FPG < 7.0 mmol/L while 2hPG ≥7.8 mmol/L, but <11.1 mmol/L, and the IFG+IGT should meet the two states above meanwhile.

### 2.6 Inclusion criteria

The participants will have to meet the diagnostic criteria of prediabetes in the Expert Consensus on Intervention of Adult Prediabetes in China, and fulfill the following conditions: they will have to meet the diagnostic criteria for obesity or overweight. They must be above the age of 18. Their Score of the American Diabetes Association Diabetes Risk Self-Test Questionnaire must be ≥25 points. They must have no other acute or chronic diseases. They must be failure in diet and exercise efforts in the early stage. They must voluntarily accept massage treatment. They must have the ability to use smart phones and social media platforms. They will have to sign the informed consent and accept the lifestyle intervention pushed by the WeChat public platform.

### 2.7 Exclusion criteria

However, the subjects will not be able to participate in this trial, if they meet any of the below indicated criteria: Patients who have been diagnosed with diabetes or other endocrine diseases, such as thyroid disease being treated. Patients who have been diagnosed with acute cardio-cerebrovascular events or myocardial infarction within 6 months. Patients who have severe impairment of liver and kidney function (Alanine transaminase, Aspartate Aminotransferase >2 times the upper limit of normal value, abnormal serum creatinine). The patient was in ketosis or hyperosmolar state. The patient was unable to exercise or required to have a special diet due to physical illness. Patients who was withdrawal, refusal of treatment, and unwillingness to exercise diet intervention during treatment. Patients who have uncontrolled hypertension (systolic blood pressure ≥160 mm Hg, diastolic blood pressure ≥110 mm Hg). Patients who have participated in similar studies or plan to undergo surgery to lose weight. Patients who are pregnant.

### 2.8 Termination of the standard

After enrollment, the trial will be effectively terminated if one of the following symptoms occurs: the patients suffer from the severe side reactions that may occur during the course of the study (medical certificate issued by the observing doctor), and severe complications or deterioration of the subjects during the study period. The subjects request to willingly withdraw from the clinical study halfway. Patients who may be uncooperative and have poor compliance. In addition, if the conditions for the termination of the test must be met after being processed by the competent doctor in accordance with the standardized working procedure, the test will be terminated immediately. The researchers will keep a detailed record of the reasons and time of withdrawal of the participants from the study. Patients with the untreated time exceedingly at least 1/2 course of treatment will be able to enter the efficacy statistics and must properly maintain a random medical record number.

### 2.9 Stripping and shedding criteria

After enrollment, patients who meet one of the following criteria will be terminated from the trial. The stripping criteria: those who might fail to meet the inclusion criteria and have entered the study. Those who might to follow the prescribed treatment or incomplete data affecting the efficacy evaluation and safety evaluation. Those who might have used the different therapeutic methods prohibited by this protocol. Those who might change the treatment plan midway by themselves. The shedding criteria: the subjects with poor compliance and self-withdrawal during the course of treatment. Those who might display serious adverse events or in those in which complications might occur. Those who will be unable to continue the treatment for various reasons.

### 2.10 Treatment programs

After being enrolled, 40 individuals will be treated with AM and healthy lifestyle intervention, with the other 40 individuals receiving only healthy lifestyle intervention. The main viscera massage techniques include rubbing the abdomen, kneading abdomen, vibrating abdomen, and pressing the abdomen. The main points to be considered will be Zhongwan (CV12), Shenque (CV8), Zhongji (CV3). The acupoint location that will be used refers to the 2006 National Standard of the People's Republic of China (GB/T12346-2006) “Acupoint Name and Location.” During the treatment, room temperature will be kept appropriate, with the environment quiet and tidy. The patient will be laid on their back, exposing the abdomen in a relaxed state. The doctor will sit on the right side of the patient. Firstly, the doctor will rub the entire abdomen clockwise with CV8 as the center, up to CV12, down to CV3, the left and right sides to the left and right meridians of the foot Taiyin Spleen meridian respectively, for 5 minutes. Next, the whole abdomen will be kneaded with both hands for 10 minutes. Then, CV12 will be vibrated with one hand with a frequency of >200 times per minute for 10 minutes. Then abdomen will be pressed with one hand on CV8, deeply while breathing in and gently while breathing out, for 5 minutes. Each treatment will last 30 minutes.

Lifestyle interventions will include diet, exercise, and other lifestyle advice and will be performed by combining pushing healthy lifestyle guidance information through Wechat application and giving face-to-face advice together daily.

All patients will receive the treatment once a day, with 2 days off every 5 days. The intervention will be conducted for 4 weeks.

### 2.11 Evaluation indicators

#### 2.11.1 Body composition measurements

Weight, BMI, waist circumference (WC), WHR, and WHtR will be measured by a special study nurse at the first day of the whole intervention and last day of every week. BMI was calculated using body weight and height measured with bare feet and in minimal clothing using a stadiometer and an electronic scale. Waist circumference will be measured at the body midsection (iliac crest), which is between the lowest point of the ribs and the highest point of the pelvis, while the participants were in a calm, exhaling position, with their legs 25 to 30 cm apart. Hip circumference will be measured at the horizontal circumference of the rear most prominent part of the hip. WHR will be calculated by WC/hip circumference. WHtR will be calculated by WC/height.

#### 2.11.2 Blood sample

Blood samples will be taken from each participant twice—one before the first treatment, and the other following the last treatment. Measurements will include triglycerides, total cholesterol, low-density lipoprotein cholesterol, and high-density lipoprotein cholesterol, FBG, 2hPG, and HbA1c, fasting insulin and HOMA-IR.

#### 2.11.3 Fecal samples

Fecal samples from the participants will be obtained before and after the intervention. Samples will be collected using the special feces collection kit. All fecal specimens will be stored in the fridge at –80 °C before and after the intervention, and then will be performed for 16S rRNA gene sequencing and metagenomic detection.

#### 2.11.4 Quality control

The study quality management will be strictly carried out with reference to the Quality Management Standard for Drug Clinical Trials. The viscera massage therapy will be performed by special well-trained chiropractors. To ensure the time, intensity, and frequency of manipulation operations be relatively consistent and meeting the clinical standardization, a thorough training, practice, and assessment of the manipulation operation will be conducted before the start of the treatment.

#### 2.11.5 Statistical method

SPSS23.0 statistical software will be used for statistical analysis for all the data generated. Chi-square test will be used for enumeration data. Paired *t* test will be used for the comparison of measurement data before and after the treatment. One-way analysis of variance (ANOVA) will be used for comparison between the groups, with *P* < .05 as difference and *P* < .01 as significant difference.

## 3 Discussion

Prediabetes is defined as blood glucose levels above normal but below diabetes thresholds, with hyperglycemia and insulin resistance the major threads.^[7]^ Prediabetes includes IFG, IGT, and IFG+IGT. The IFG subjects always have high hepatic insulin resistance with almost normal values in skeletal muscle, while high insulin resistance in skeletal muscle with only modest changes in liver insulin sensitivity is commonly in IGT subjects.^[3]^ The incidence of prediabetes is rising as the prevalence of obesity increases globally.^[7]^ So, in this trial, we will select not only the common indicators of prediabetes, such as FBG, 2hPBG, and HbA1c, but also the indicators associated with obesity, like weight, BMI, WC, WHR, WHtR, and blood lipid level. And studies have found that a combination of WC and WHtR may be a better clinical utility than BMI and WHR in identifying individuals with cardiometabolic risk factors.^[21]^ Fasting insulin and HOMA-IR will be used to test the level of insulin resistance. Through the proposed clinical trial, the effect of AM on prediabetes and obesity will be further verified.

Prediabetes, diabetes, and obesity are worldwide epidemics, with an abundance of evidence pointing to the link between gut microbiome and these disorders.^[22,23]^ The key role of the gut microbiota in the progression and prevention of metabolic associated disorders is becoming increasingly apparent.^[24]^ Abnormalities in gut microbiota might contribute to the disturbed energy and substrate metabolism and obesity-related chronic low-grade inflammation, which exactly the cause of the prediabetes, diabetes, and obesity-related diseases.^[25]^ A lot of studies have found lifestyle interventions, as basic treatment therapy, can alleviate prediabetes, diabetes, and obesity-related diseases through regulating disturbed gut microbiome.^[26–28]^ The practice of viscera massage on prediabetes, diabetes, and obesity-related diseases also achieved good effect, but whether the underlying mechanisms affect gut microbiota is unclear.^[11,29]^ Viscera massage therapy not only be used to treat metabolic diseases, but also be used more widely to treat digestive system diseases, especially constipation. Studies have found that viscera massage can ease constipation by reducing colonic transport time (CTT) and promoting intestinal motility.^[30,31]^ Meanwhile, the relationship of CTT and human colonic metabolism and its importance for host health came under observation. Research showed stool consistency is strongly associated with gut microbiota richness and composition, enterotypes, and bacterial growth rates.^[32]^ The increased CTT has been linked to enhanced proteolytic fermentation and associated production of potentially deleterious metabolites.^[32]^ Another study found that the diversity and richness of gut microbiota correlated much more strongly with CTT than with other phenotypic measures including waist circumference, triglyceride levels, and blood pressure.^[33]^ Since AM can promote intestinal motility, and change of CTT can modulate gut microbiota, we can infer that AM can modulate composition of gut microbiota affects human metabolism. So, in this trial, we will explore the mechanism of AM on prediabetes from the perspective of intestinal microbiota. The fecal samples of subjects will be performed 16S rRNA gene sequencing and metagenomic detection. We hope the reason of viscera massage to alleviate prediabetes, obesity, and other diseases will be discovered.

## 4 Trial status

On February 1, 2021, the medical Ethics Committee of Affiliated Hospital of Changchun University of Traditional Chinese Medicine approved the study plan (CCZYFYLL2021 Standard word-002). Due to the delay in recruitment and preparation time, the trial run will begin in December 2021, and we expect the recruitment of the volunteers to be completed by February 2023.

## 5 Advantages and limitations of proposed study

The study will be safe and reliable. We will perform abdominal massage and lifestyle modifications on patients with prediabetes. The 2 types of intervention means are almost no risk.

After treatment, the individuals will be assessed by physical examination (weight, BMI, WC, WHR, WHtR), chemical examination (triglycerides, total cholesterol, low-density lipoprotein cholesterol, high-density lipoprotein cholesterol, FBG, 2hPG and HbA1c, fasting insulin, and HOMA-IR), and 16S rRNA gene sequencing and metagenomic detection, the above of which are harmless to people.

The advantages of this study will be its safety, reliability, practicability. In this clinical trial, the rights and interests of the subjects will be fully guaranteed. The limitation is the lack of double-blind control and completely-randomized design.

## Author contributions

**Conceptualization:** Deyu Cong, Xingquan Wu.

**Funding acquisition:** Yunjie Chu, Xingquan Wu.

**Investigation:** Hongyu Ju, Jiabao Sun, Chaozheng Li, Zepeng Zhang.

**Methodology:** Gaofeng Wang, Xingquan Wu.

**Project administration:** Gaofeng Wang, Jiabao Sun.

**Resources:** Gaofeng Wang, Xingquan Wu.

**Writing – original draft:** Xingquan Wu.

**Writing – review & editing:** Gaofeng Wang.
